# Hematopoietic Stem Cell Transplantation in an Infant with Immunodeficiency, Centromeric Instability, and Facial Anomaly Syndrome

**DOI:** 10.3389/fimmu.2017.00773

**Published:** 2017-06-30

**Authors:** Katharina L. Gössling, Cyrill Schipp, Ute Fischer, Florian Babor, Gerhard Koch, Friedhelm R. Schuster, Jutta Dietzel-Dahmen, Dagmar Wieczorek, Arndt Borkhardt, Roland Meisel, Michaela Kuhlen

**Affiliations:** ^1^Medical Faculty, Department of Pediatric Oncology, Hematology and Clinical Immunology, University of Düsseldorf, Düsseldorf, Germany; ^2^Department of Pediatrics, Allgemeines Krankenhaus Hagen, Hagen, Germany; ^3^Medical Faculty, Department of Human Genetics, University of Düsseldorf, Düsseldorf, Germany

**Keywords:** immunodeficiency, centromeric instability, facial anomaly, ICF syndrome, agammaglobulinemia, hematopoietic stem cell transplantation, *Pneumocystis jirovecii* pneumonia

## Abstract

Immunodeficiency, centromeric instability, and facial anomaly (ICF) syndrome is a rare autosomal recessive genetic condition with severe immunodeficiency, which leads to lethal infections if not recognized and treated in early childhood. Up-to-date treatment regimens consist of prophylactic and supportive treatment of the recurrent infections. Here, we report the case of a 1-year-old boy of Moroccan consanguineous parents, who was diagnosed at 4 months of age with ICF syndrome with a homozygous missense mutation in the *DNMT3B* gene. He was initially admitted to the hospital with recurrent pulmonary infections from the opportunistic pathogen *Pneumocystis jirovecii (PJ)*. Further immunological workup revealed agammaglobulinemia in the presence of B cells. After successful recovery from the PJ pneumonia, he underwent hematopoietic stem cell transplantation (HSCT) from the HLA-matched healthy sister using a chemotherapeutic conditioning regimen consisting of treosulfan, fludarabine, and thiotepa. Other than acute chemotherapy-associated side effects, no serious adverse events occurred. Six months after HSCT immune-reconstitution, he had a stable chimerism with 2.9% autologous portion in the peripheral blood and a normal differential blood cell count, including all immunoglobulin subtypes. This is one of the first cases of successful HSCT in ICF syndrome. Early diagnosis and subsequent HSCT can prevent severe opportunistic infections and cure the immunodeficiency. Centromeric instability and facial anomaly remain unaffected. Although the long-term patient outcome and the neurological development remain to be seen, this curative therapy for immunodeficiency improves life expectancy and quality of life. This case is meant to raise physicians awareness for ICF syndrome and highlight the consideration for HSCT in ICF syndrome early on.

## Introduction

Immunodeficiency, centromeric instability, and facial anomaly (ICF) syndrome is a rare autosomal recessively inherited genetic condition. The majority of the affected individuals have mutations in the methyltransferase 3B gene (*DNMT3B*, OMIM 602900) on chromosome 20 leading to reduced DNA methylation of the pericentromeric regions of chromosomes 1, 9, and 16 (1, 2). Epigenetic dysregulation rather than a single gene defect determines the clinical phenotype. Although all body cells carry the same mutation, various tissues are differently affected due to varying degrees of DNA methylation. This is especially seen in mitogen-stimulated lymphocytes where whole arm deletions, translocations, and multibranched chromosomes cause an abnormal gene regulation of B cell immunoglobulin isotype switching, lymphocyte activation, and migration (3). ICF patients suffer from recurrent gastrointestinal and pulmonary infections in early childhood due to the agammablobulinemia resulting in failure to thrive (4). An intrinsic T cell defect has also been linked to the high frequency of opportunistic infections from pathogens such as with *Pneumocystis jirovecii (PJ)*, but the exact mechanism has not been elucidated (5). Typical clinical characteristics include the eponymous facial anomaly of epicanthic folds, hypertelorism, and a flat nasal bridge, as well as a delay in psychological and cognitive development.

Treatment options are limited and consist primarily of supportive therapy such as substitution of immunoglobulins, prophylactic sulfamethoxazole–trimethoprim therapy, or antibiotic therapy (6–8). Life expectancy of ICF patients is poor and prognosis is dependent on the frequency and severity of infections. A high proportion of reported ICF patients die at a young age (9). Early IgG replacement and antibiotic prophylaxis can significantly improve patient outcomes (10). An early sustainable therapy for the immunodeficiency can dramatically better the disease course. The only curative treatment of the immune dysfunction is hematopoietic stem cell transplantation (HSCT), as described in isolated case studies (3, 7). However, no long-term follow-up data are available to date.

## Background

Here, we report the case of a 1-year-old boy of Moroccan consanguineous descent diagnosed with ICF syndrome carrying a homozygous missense mutation in the *DNMT3B* gene (Ala603Thr) with hypogammagobulinemia, normal B cell count, facial anomaly, and failure to thrive.

This variant has been previously described in ICF syndrome by Hansen et al. It consists of a point mutation (rs121908943) and an amino acid exchange from alanine to threonine. The variant has a minor allele frequency of *A* = 0.000008 (11) and a prevalence of less than 1/1,000,000. Hansen et al. described this variant among other mutations in the DNMT3B gene, while 105 control DNAs from unrelated healthy Caucasians in North America and the Netherlands did not carry this mutation (1).

**Figure 1:**
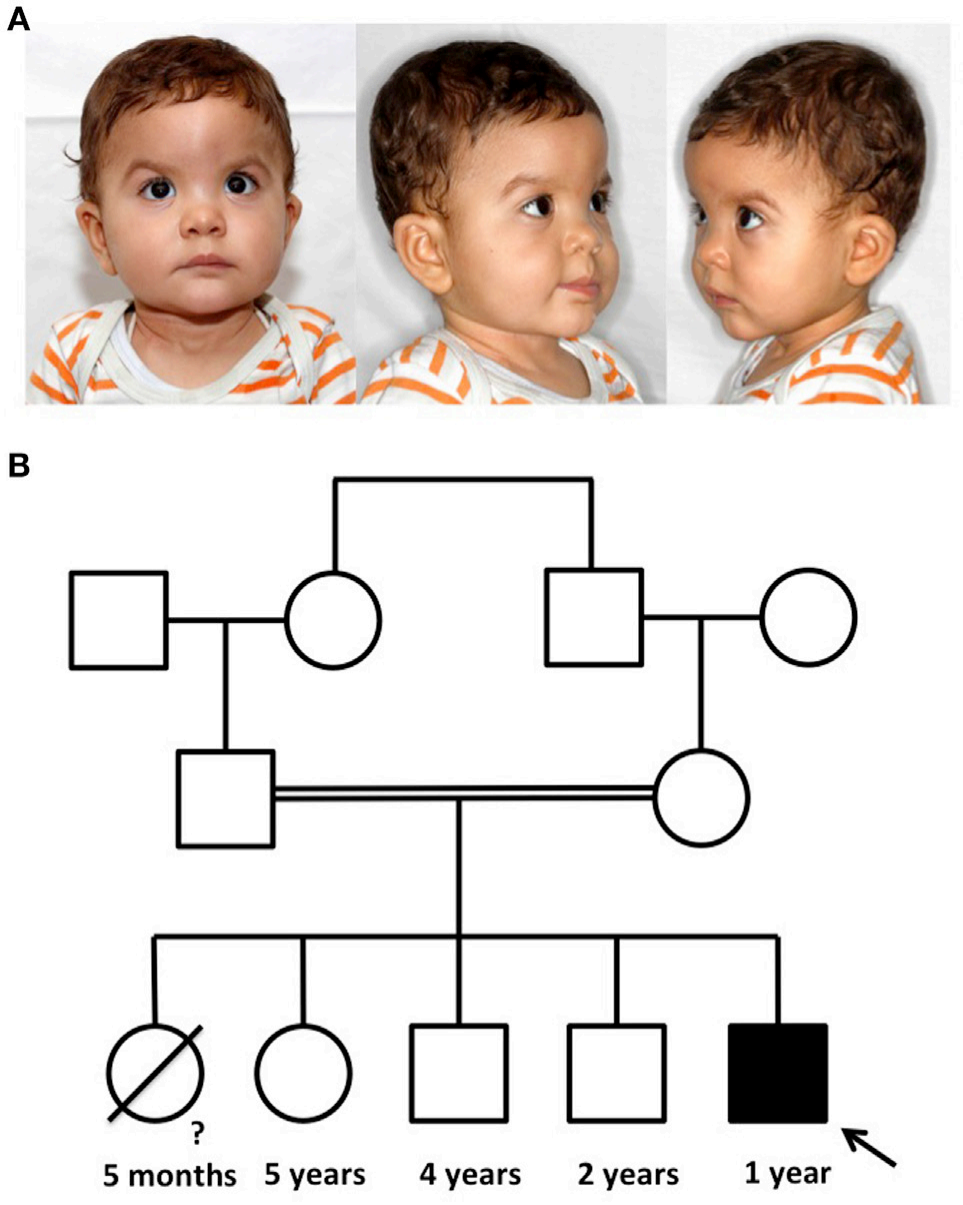
Picture of the patient with immunodeficiency, centromeric instability, and facial anomaly (ICF) syndrome and pedigree of the family. **(A)** The typical phenotypic characteristics for ICF syndrome are epicanthic folds, telecanthus, hypertelorism, a flat nasal bridge, and low-set and posteriorly rotated ears. **(B)** The patient is the fifth child of consanguineous parents. The first daughter died at the age of 5 months from unknown causes. Three older siblings are healthy.

**Figure 2:**
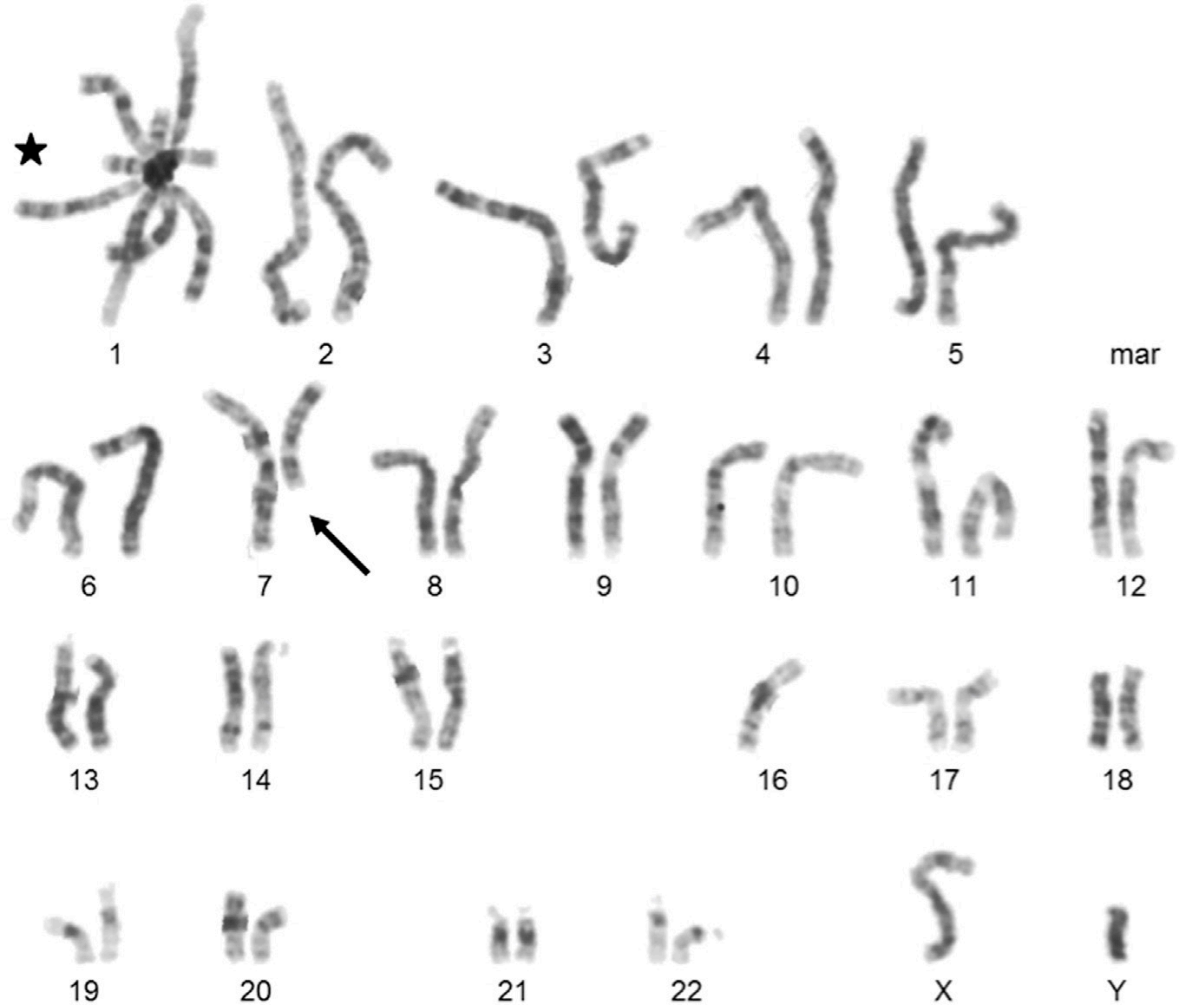
Karyogram of the cytogenetic analysis. This metaphase shows formation of a multiradial figure (indicated by a star), a terminal deletion of a part of the long arm of chromosome 7 (indicated by an arrow) and a loss of one chromosome 16.

The boy was born with a body weight of 3,200 g (38th percentile) and length of 51 cm (72th percentile) (CDC/WHO growth charts 2000). At the age of 2.5 months, his body weight was at 5,300 g (12th percentile) and the length was 59 cm (24th percentile) (CDC/WHO growth charts 2000). At the age of 4 months, the patient was admitted to the hospital for the first time with recurrent and prolonged respiratory infections of the lower and upper airways with hypoxemia. Laboratory workup revealed a bilateral bronchopneumonia positive for *Parainfluenza* virus type 1 and 3 and *PJ*, as well as purulent conjunctivitis and purulent otitis media perforans positive for *Haemophilus influenzae*. In the subsequent immunological diagnostics, a significantly reduced level of all immunoglobulin subclasses (Table 1) and a normal B cell count with a lack of memory B cells were detected. T cell count and proliferation rate of CD4+ and CD8+ cells after mitogenic stimulation with phytohemagglutinin were normal. Bronchoscopy revealed normal anatomic proportions of the airways. The *PJ* pneumonia was treated with intravenous cotrimoxazole, corticosteroids, and oxygen supplementation. Subsequent regular intravenous immunoglobulins treatment and prophylactic cotrimoxazole prevented further severe pulmonary infections. In addition, the patient has typical facial dysmorphisms consisting of hypertelorism, flat nasal bridge, epicanthic folds, and low set ears (Figure 1A—pictures of the face). ICF syndrome was clinically suspected and confirmed with the cytogenetic analysis revealed whole arm deletions, translocations, and multibranched chromosomes due to the centromeric instability in chromosomes 1, 9, and 16 (Figure 2—karyogram). The patient is the fifth child of consanguineous Moroccan parents, who are first cousins (Figure 1B—pedigree). Three healthy older siblings (5-, 4-, and 2-year olds) developed normally. ICF in the siblings was excluded with normal blood levels of immunoglobulins and a normal karyogram. The oldest daughter of the family died of respiratory failure at the age of 5 months in Morocco, no further genetic or pathological diagnostics were performed *post mortem*.

**Table 1:** Immunological parameters before and 6 months post-hematopoietic stem cell transplantation (HSCT).

	Before HSCT	6 months post HSCT
IgG (mg/dl)	52	571
IgA (mg/dl)	<5	57
IgM (mg/dl)	<5	51
CD4+ (c/μl)	1,840	818
CD8+ (c/μl)	450	613
CD4+/CD8+	4.1	1.3
CD20+ (c/μl)	1,329	588
CD20+IgD+CD27− (c/μl)	1,329	588
CD20+IgD−CD27+ (c/μl)	0	7
CD56+ (c/μl)	208	148
CD14+ (c/μl)	913	292
CD66b+ CD49d− (c/μl)	2,040	3,723
Chimerism (BM) (% autologous portion)	–	1–5
Chimerism (blood) (% autologous portion)	–	2.9

*Immunoglobulins (Ig) G, A, A were measured in the serum by ELISA. T helper cells (CD4+), cytotoxic T cells (CD8+), B cells (CD20+), naïve B cells (CD20+IgD+CD27−), memory B cells (CD20+IgD− CD27+), NK-cells (CD56+), monocytes (CD14+), neutrophilic granulocytes (CD66b+CD49d−) were measured by flow cytometry. Chimerism analysis in bone marrow (BM) and blood was performed using molecular genetic methods or XY-Fish, respectively*.

**Figure 3:**
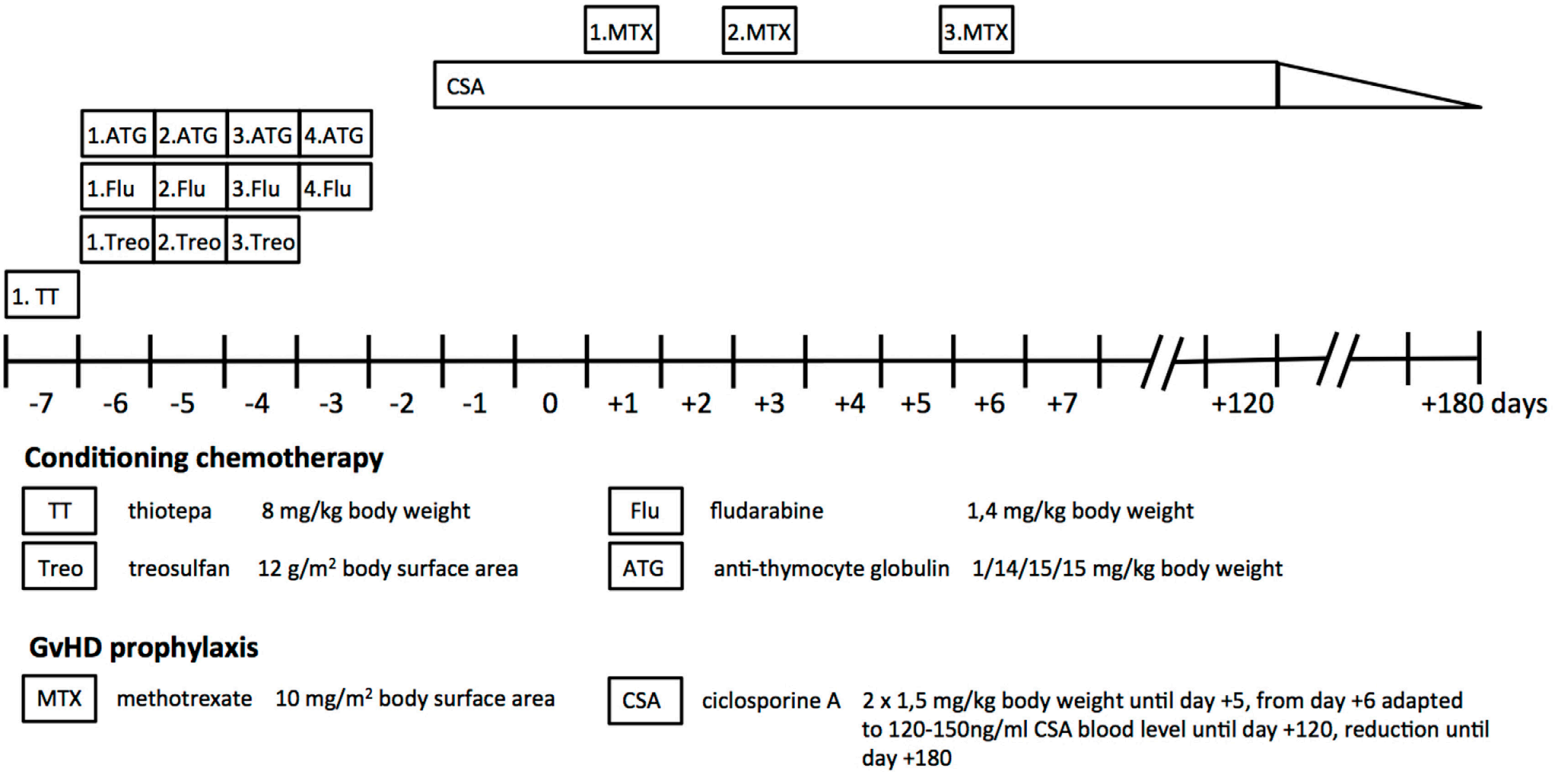
Treatment regimen. The myeloablative conditioning chemotherapy before transplantation consisted of thiotepa, fludarabine, treosulfane, and antithymocyte globulin. Cyclosporine A and methotrexate were given as immunosuppressive prophylaxis for graft-versus-host disease (GvHD) after transplantation.

At the age of 5 months, he presented with a body weight of 6,600 g (8th percentile) and a length of 66 cm (37th percentile), failure to thrive was diagnosed (CDC/WHO growth charts 2000). At the age of 6 months, the patient underwent hematological stem cell transplantation from the healthy 5-year-old sister with a HLA match of 10/10 as donor. After the conditioning regimen consisting of thiotepa, treosulfan, and fludarabine, he received CD34+ cells (24.7 × 10^6^ cells/kg bodyweight). Cyclosporine A, methotrexate, and antithymocyte globulin were given as prophylaxis for graft-versus-host disease (GvHD) (Figure 3). Patients with ICF syndrome often display residual T cell function that may result in graft failure or rejection and are, therefore, subjected to reduced intensity conditioning (3). Given the good performance status of our patient, a myeloablative conditioning with reduced toxicity was administered in order to achieve stable lymphohematopoietic engraftment with low risk of organ toxicity (12).

He suffered from acute chemotherapeutic reactions in the mucosa (toxicity WHO grade 4) and on the skin (toxicity WHO grade 1), nausea and vomiting (WHO grade 2), and renal clearance impairment (WHO toxicity grade 2). With time, he recovered completely from all side effects. Leukocyte and neutrophil engraftment was achieved on day +21 and on day +22, respectively. The chimerism analysis of the blood showed an autologous portion at a maximum of 0.5%, while the chimerism of the bone marrow was completely from the donor. Without any infections or signs of GvHD, he was discharged from the hospital on day +47. He continued to receive cyclosporine A until day +120, which was stepwise reduced in the absence of any signs of GvHD. Three months after transplantation, he was admitted again to the hospital with RSV infection that was successfully treated with Ribavirin. Four months posttransplantation, he was admitted again due to diarrhea, obstructive bronchitis, and fever. Antibiotic treatment was started initially and discontinued when influenza, RSV, and corona virus were detected in the throat. Without specific antiviral therapy, he recovered completely. Five months after transplantation, he presented with diarrhea, subfebrile temperature, and a partially compensated metabolic acidosis. Rotavirus was detected in the stool. IV-fluid therapy as an outpatient was sufficient to normalize the blood gases and the symptoms receded spontaneously. Six months after transplantation, adenovirus was detected in blood, stool, and throat without any clinical symptoms. An intranvenous treatment with cidofovir was started until the virus cleared sufficiently.

His differential blood counts increased steadily and were normal 6 months after transplantation (Table 1). The chimerism in the bone marrow showed an overall autologous portion of 1–5%, with 1–5% in the CD3+ and CD33+ fractions as well as 0–1% in the CD34+ subset. The chimerism in the blood was with 2.9% autologous cells stable over the last 4 months.

At 1 year of age, his neurological development correlated approximately with a 7-month-old child. His body weight was 8,590 g (9th percentile) and the length 75 cm (18th percentile) (CDC/WHO growth charts 2000).

## Discussion

We report the successful allogeneic HSCT of a 1-year-old boy with ICF1 syndrome carrying a homozygous mutation in the *DNMT3B* gene after receipt of bone marrow cells from the 10/10 HLA-matched clinically healthy sister. During the 1-year follow-up, we observed a complete immune reconstitution without acute or short-term transplantation-related complications.

Immunodeficiency, centromeric instability, and facial anomaly syndrome is a rare and severe immunodeficiency disease. Our patient is the fifth child of consanguineous parents and underwent HSCT at the early age of 6 months. The first daughter of the same parents died at 5 months of age in 2009 in Morocco due to respiratory failure of unknown origin. Since no further diagnostics were conducted *post mortem*, we can only speculate that the same homozygous mutation in the *DNMT3B* gene might have been the cause for her early death. While a gender bias for ICF2 syndrome caused by a mutation in the *ZBTB24* gene has been observed recently, ICF1 syndrome affects both genders equally making it even more plausible that the deceased daughter had the same genetic defect as our patient (13).

This family history emphasizes the fact that ICF patients have very poor clinical outcomes if they are not treated early and adequately. In case reports from the 1990s, only patients on Ig therapy survived to childhood, while others died within the first year of life from opportunistic infections (9) as described in nine ICF cases in France. Recently, Gennery et al. published the successful cure of the immunodeficiency by HSCT in three ICF patients, who received HSCT at the ages of 18 months, 2 and 4 years (3). Here, we report another case of successful HSCT in ICF syndrome that received HSCT at 6 months of age. HSCT itself is associated with a high risk of mortality due to short-term opportunistic infections and GvHD. Long-term side effects include chemotherapeutic toxicity such as liver or kidney dysfunction, growth retardation, infertility, and secondary malignancies (14). Patients who had never received chemotherapeutic treatment beforehand usually have a better clinical outcome and can be transplanted successfully with few acute side effects as seen in patients with hemoglobinopathies or sickle-cell anemia (15, 16).

Since severe infections significantly worsen patient outcomes after HSCT, we aim to perform HSCT in ICF syndrome as early as possible in an infection-free period. ICF syndrome is invariably associated with low or absent levels of immunoglobulins given the defective peripheral terminal B cell differentiation. Indeed, this could be treated with life-long IgG replacement. However, a number of these patients also display functional T cell immunodeficiency, as evidenced in our case by *PJ* pneumonia. Furthermore, recent studies from Rechavi et al. and Weemaes et al. highlight the fact that a T cell proliferation defect develops over time (4, 17). As such, patients with ICF syndrome suffer from multisystem disease leading to long-term morbidity and substantial mortality. This usually stems from severe infections (i.e., in the GI tract) associated with growth retardation and limited life expectancy. Particularly in an advanced stage of the disease, HSCT is associated with a higher risk of mortality due to preexisting infections. Allogeneic HSCT is, therefore, an attractive therapeutic approach in those patients with signs of T cell immunodeficiency without severe preexisting morbidity and is usually recommended when an HLA-matched donor can be identified. Although the conditioning chemotherapy is accompanied by high toxicity, we believe the advantages of HSCT significantly outweigh the natural disease course in ICF. HSCT replaces all hematopoietic cells carrying the *DNMT3B* mutation and can thereby cure the immunodeficiency in ICF patients. Weemaes et al. and Hagleitner et al. examined the clinical features of ICF1 patients, which can help to predict clinical outcomes after HSCT (4, 7). Recurrent infections and a higher risk for hemato-oncological malignancies are eliminated in our patient with HSCT, while a delayed neurological development or cortical atrophy with seizures are features that remain (4, 7). During the immunosuppressive treatment and before reconstitution of the adaptive immune system, our patient suffered from recurrent respiratory and gastrointestinal infections. This can be attributed to the posttransplantation immunosuppression and are expected to improve with time. While failure to thrive can be attributed to the high frequency of infections, we expect our patient to catch up on weight gain within the coming months.

DNA methylation is a dynamic process that epigenetically regulates gene expression during development and other processes such as aging, cancerogenesis, or cell differentiation. The *DNMT3B* mutation was described to lead to altered epigenetic modifications of genes regulating development, neurogenesis, and immune function (18). Since the DNA methyltransferase is an intracellular enzyme that is not secreted and taken up by the surrounding tissue, HSCT is not able to compensate for the enzyme deficiency in all other cell types of the body. A recent study shows next to the hypomethylation of the pericentromeric regions of chromosomes 1, 9, and 16, a global loss of methylation in ICF patients’ fibroblasts and lymphoblastoid cell lineages leads to significantly upregulated gene expression of the differentially methylated positions (19). Although 181 of such regions were identified, it remains unclear, how and to which degree a given tissue is affected by the hypomethylation and to what extent this pattern changes during development. All these questions need to be answered before we are able to translate the molecular findings into the clinical phenotype. Nevertheless, we can speculate that an overactive gene expression might have a great impact on genes that are highly epigenetically regulated, such as those responsible for neurological development or germ line genes important in preventing cancer development. Both of which can still lead to delayed neurological development and a higher risk for malignancies in ICF patients even after successful HSCT.

## Concluding Remarks

Seeing the combination of a mild facial abnormality, agammaglobulinemia in the presence of normal B cell count and opportunistic infections in a patient, physicians must consider ICF syndrome as a differential diagnosis. HSCT is the only curative treatment option of the immune defect and should be carried out early to improve survival. However, developmental and neurological outcomes remain largely unchanged since the epigenetic dysregulation of non-hematopoetic cells cannot be corrected with HSCT.

## Ethics Statement

The study was approved by the local ethics committee and carried out in accordance with the Declaration of Helsinki. The parents provided written informed consent to publish the report and the picture appearing in Figure 1.

## Author Contributions

KG first drafted the manuscript. CS and UF contributed to WES analysis. AB, FB, FS, GK, and RM cared for the child. JD-D and DW performed cytogenetic analysis. AB cared for the child and critically revised the manuscript for important intellectual content. MK cared for the child, designed, and supervised the project and critically reviewed and revised the manuscript for important intellectual content. All authors approved the final manuscript as submitted.

## Conflict of Interest Statement

The authors declare that the research was conducted in the absence of any commercial or financial relationships that could be construed as a potential conflict of interest.
